# Serum Tumour Markers in Testicular Germ Cell Tumours: Frequencies of Elevated Levels and Extents of Marker Elevation Are Significantly Associated with Clinical Parameters and with Response to Treatment

**DOI:** 10.1155/2019/5030349

**Published:** 2019-05-28

**Authors:** Klaus-Peter Dieckmann, Hanna Simonsen-Richter, Magdalena Kulejewski, Petra Anheuser, Henrik Zecha, Hendrik Isbarn, Uwe Pichlmeier

**Affiliations:** ^1^Asklepios Klinik Altona, Abteilung für Urologie, Hodentumorzentrum Hamburg, Hamburg, Germany; ^2^Albertinen-Krankenhaus Hamburg, Klinik für Urologie, Hamburg, Germany; ^3^Universitätsklinikum Eppendorf, Hamburg, Martini Klinik, Hamburg, Germany; ^4^Universitätsklinikum Eppendorf, Hamburg, Zentrum für Experimentelle Medizin, Institut für Medizinische Biometrie und Statistik, Hamburg, Germany

## Abstract

**Introduction:**

Although serum tumor markers beta human chorionic gonadotropin (bHCG), alpha-fetoprotein (AFP), and lactate dehydrogenase (LDH) are well-established tools for the management of testicular germ cell tumours (GCTs), there are only few data from contemporary cohorts of primary GCT patients regarding these biomarkers. Our aim was to evaluate marker elevations in testicular GCTs and to document their associations with various clinical characteristics.

**Patients and Methods:**

A total of 422 consecutive patients with GCTs were retrospectively analysed regarding serum levels of bHCG, AFP, and LDH during the course of treatment. Additionally, the following characteristics were recorded: histology, age, laterality, clinical stage (CS), pT-stage, and tumour size. Marker elevations were first tabulated in dichotomized way (elevated: yes/no) in various subgroups and second as continuous measured serum values. Descriptive statistical methods were employed to look for differences among subgroups and for associations of elevations with clinical parameters.

**Results:**

In all GCT patients, the frequencies of elevated levels of bHCG, AFP, LDH, and bHCG or AFP were 37.9%, 25.6%, 32.9%, and 47.6%; in pure seminomas 28%, 2.8%, 29.1%, and 30.3%; and in nonseminoma 53.0%, 60.1%, 38.7%, and 73.8%. Significant associations were noted with pT-stages >pT1, clinical stages >CS1, tumour size, and younger age. Frequencies of marker elevations dropped significantly after treatment, but LDH levels remained elevated in 30.5%-34.1%. Relapsing patients (n=27) had elevated levels of bHCG, AFP, and LDH in 25.9%, 22.2%, and 29.6%, respectively, thirteen of whom with a changed marker pattern.

**Conclusions:**

The classical GCT-biomarkers correlate with treatment success. Clinical utility is limited due to proportions of < 50% of patients with elevated levels and the low specificity of LDH. The elevation rates are significantly associated with histology, clinical and pT-stages, tumour size, and younger age. Individual marker patterns may change upon relapse. Clinically, ideal biomarkers are yet to be found.

## 1. Introduction

Serum tumour markers alpha fetoprotein (AFP), beta human chorionic gonadotropin (bHCG), and lactate dehydrogenase (LDH) represent valuable tools for the clinical management of testicular germ cell tumours (GCTs) [1]. They were first introduced into clinical practice in the 1970s [2, 3] and became international standard tools with the world wide implementation of the immunologically based ELISA measurement technique [4]. According to current guidelines, serum tumour markers are used to assist timely diagnosis of GCT, to accurately stage the disease, to assess the prognostic category of metastasized GCTs, to monitor treatment success, and finally to detect relapse during follow-up [5, 6]. bHCG is a 38 kDa glycoprotein produced by syncytiotrophoblastic giant cells mainly in chorionic carcinoma [7]. AFP is a 70 kDa glycoprotein produced by cells of the yolk sac tumour and rarely by embryonal carcinoma [8, 9]. LDH is a glycolytic enzyme that is present in all cells of the human body and that is released from cells upon cell death. Due to its unspecific origin, the clinical usefulness of LDH is less than that of the other two markers [10, 11]. Clinical data relating to the three markers were mostly generated in the last century [2, 3], subsequently reviewed, and finally included into guidelines [12]. Based on the biological diversity of GCTs, it was early recognized that not all GCTs have elevations of these markers [13] and that the frequencies of elevation correlate with histology and tumour burden [4, 14, 15]. A recent meta-analysis revealed a prevalence rate of LDH in 40-60% of all GCT cases [10]. AFP is exclusively found to be elevated in 10-60% of nonseminomatous GCTs. BHCG is elevated in 10-40% of nonseminomas and in 15-20% of seminomas while prevalence rates apparently depend on clinical stages [16]. Surprisingly few original data are available relating to contemporary patient cohorts, also the associations of marker positivity with clinical parameters is incompletely understood. Therefore, the aim of the present study was to perform a survey on the frequency of tumour marker elevations in a large contemporary cohort of unselected primary GCT patients and to analyse the associations of marker positivity with various clinical characteristics.

## 2. Patients and Methods

The charts of 422 consecutive patients of European ancestry undergoing treatment for testicular GCT at Albertinen-Krankenhaus, Hamburg, during 2000–2017 were retrospectively evaluated for the serum levels of bHCG, AFP, and LDH at the following five points of time during the course of treatment: at diagnosis, after orchiectomy but before any further treatment, (if applicable) after the first cycle of chemotherapy, (if applicable) after completion of further therapy, and (if applicable) at the time of relapse. In addition, the following parameters were registered: histology of GCT (seminoma, nonseminoma), age at presentation (years), localization (left/ right side), pathological local tumour stage (pT-stage) defined as pT1 or >pT1 (pT 2-4) according to the UICC classification of 2002 [36], clinical stage (CS, Lugano classification), and size of primary tumour (cm). The control group, as previously reported, consisted of 208 patients, aged 18–55 years with other urological diagnoses except for malignant diseases [37]. All of the blood aspirations had been done during routine clinical examinations of the patients. All investigations were performed in compliance with the Helsinki Declaration of the World Medical Association (as amended by the 64th General Assembly, 2013). Ethical approval for the study was given by the institutional ethical committee (U1b/2016). All of the measurements of the serum marker levels had been performed in a clinical routine laboratory with commercially available enzyme immune assay kits. During the time span of evaluation, the provider of laboratory kits had been changed twice for economic reasons, and the upper limits of norm (ULN) thus changed accordingly. To overcome the problem of changing normal ranges during the time span of evaluation, we registered all of the individual serum levels as x-fold value of the corresponding ULN.

We addressed the following questions: at the time of diagnosis of GCT, how many patients had elevations of either bHCG, or AFP, or LDH, or any combination of the three? Are the frequencies of marker elevation and the extents of marker elevation associated with histology, age, clinical stages, pT-stages, tumour size, or localisation? How do prevalence rates and marker level extents change during the course of treatment? How many of the relapsing patients have elevated markers and is the marker pattern at relapse identical with that of first diagnosis?

### 2.1. Statistical Methods

Individual data were initially registered in a database using MS Excel software. Final analysis was performed with SAS software package version 9.4 (SAS Institute, Cary, NC, USA) on windows platform.

To assess associations of marker elevations with tumour size, we defined the following five size-categories: ≤ 1 cm; >1 -2 cm; >2 – 4 cm; >4 – 6 cm; >6 cm. Patients' ages were categorized as follows: ≤20 yrs; >20 – 30 yrs; >30 – 40 yrs; >40 – 50 yrs; > 50 yrs. Clinical stages were categorized as CS1, CS2a,b, CS2c, and CS3, and in a further analysis, into CS1 and >CS1. Each analysis comprised of two steps, first a dichotomized evaluation of the various groups with documenting if marker elevation was present or not (yes/ no; prevalence rate), and second if appropriate, the recording of serum levels (x-fold of ULN) as continuous variables (i.e., extent of marker elevation). Marker prevalence rates are reported as relative proportions with 95% confidence intervals (CIs). Comparisons of proportions were performed with the chi-square test. Comparisons of continuous variables were done with the Mann-Whitney U test and the Kruskal Wallis test in case of two and at least three groups, respectively. The McNemar Test was employed for comparisons of prevalence rates at two different time-points whereas the Friedman test was applied for time-dependent comparison of continuous variables.

## 3. Results

### 3.1. Marker Prevalence Rates, Extent of Marker Elevations, and Associations with Histology and Stages

In the entire cohort of GCT patients, the dichotomized evaluation revealed elevations of bHCG, AFP, and LDH, or elevation of any of the three markers in 37.9% (95%CI 33.3 – 42.8%), 25.6% (95%CI 21.6 – 30.1%), 32.9% (95%CI 28.5-37.7%), and 59.5% (54.6 – 64.2%), respectively. The rates of all other marker combinations and other details are given in Tables 1 and 2. Prevalence rates of bHCG and LDH were significantly higher in advanced pT-stages (>pT1). In the entire GCT population and also in both histological subgroups, prevalence rates of each of the three markers and of all combinations thereof were significantly higher in clinical stages > CS1. Localisation (left/right) was not associated with marker elevation. Prevalence rates of each of the markers were significantly higher in nonseminoma than in seminoma (see Table 2 for details). 28% of seminoma patients had elevations of bHCG. As reported previously, very few seminoma patients (2.8%) had elevations of AFP that were obviously unrelated to the malignant disease [37]. In nonseminoma patients, prevalence rates of all three markers and combinations thereof were significantly higher in advanced clinical stages (> CS1) than in localized disease. In seminoma, this was also true for bHCG and LDH.

**Figure 1:**
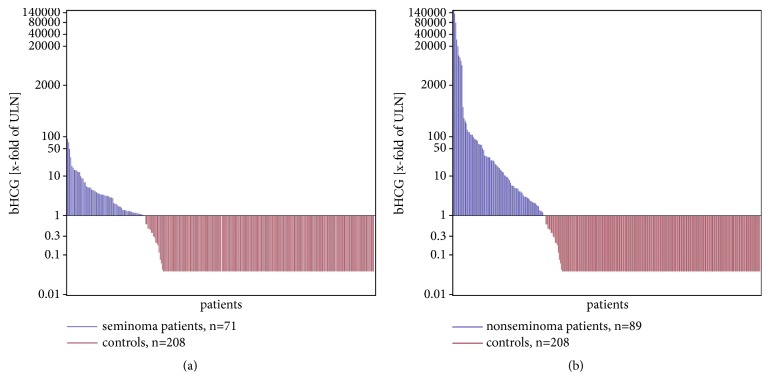
(a) Individual serum bHCG levels in controls (red) and seminoma patients (blue) ranked by the extent of elevation (x-fold of ULN). Waterfall plot: horizontal line denotes upper limit of norm (ULN). Logarithmic scale on y-axis. (b) Individual serum bHCG levels in controls (red) and nonseminoma patients (blue). The figure illustrates greatly elevated bHCG levels (>10-fold of ULN) to be rare in seminoma but frequent in nonseminoma.

**Figure 2:**
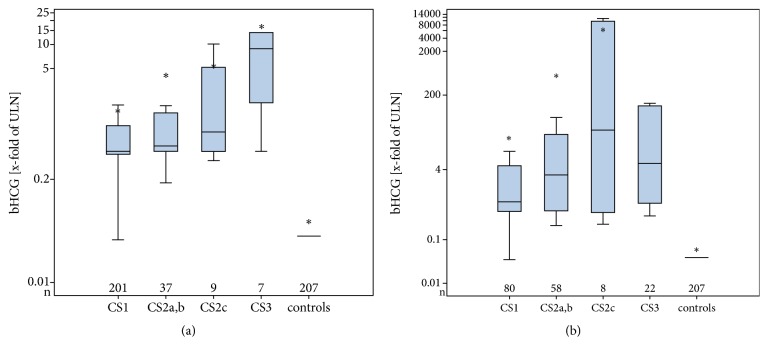
(a) Median serum levels of bHCG in various clinical stages of seminoma patients. Box plot illustration: bars within boxes denote median value. Upper and lower limits of the boxes denote upper (Q3) and lower (Q1) quartile limits, respectively. Whiskers are defined by values larger than Q3 or smaller than Q1, respectively, by at most 1.5 times the interquartile range. Stars denote mean values of groups. Logarithmic scale on y-axis. ULN: upper limit of norm. (b) Box plot illustration of median serum levels of bHCG in the various clinical stages of nonseminoma.

Median serum levels (x-fold of ULN) of both, bHCG and AFP, were significantly higher in nonseminoma than in seminoma (details in Table 3). As illustrated in Figures 1(a) and 1(b), the number of greatly elevated bHCG levels (>10x of ULN) is much higher in nonseminoma than in seminoma. Most of the bHCG elevations in seminoma represent serum values below 10-fold of ULN. Marker levels were associated with clinical stages in the cohort of all GCT patients. The levels of all three markers were lowest in CS1 and highest in CS3 with intermediate levels in CS2. Figures 2(a) and 2(b) exemplify the correlation of bHCG levels with clinical stages in seminoma and nonseminoma. The differences of median marker levels among clinical stages were statistically significant with respect to all of the three markers. Comparison of the median marker levels of CS1 patients with the median levels of all metastasized patients (>CS1) revealed significant differences regarding bHCG and LDH but not AFP in both seminoma and nonseminoma.

### 3.2. Association of Marker Elevations with Tumour Size and with Age

In the cohorts of all CS1 GCT patients (n=280) and of those with CS1 seminoma (n=201), the dichotomized evaluation revealed significantly higher prevalence rates of bHCG and LDH, respectively, with increasing tumour size (Table 4). In CS1 nonseminoma (n=79), prevalence rates of all of the three markers and of all combinations thereof significantly increased with increasing tumour size.

**Figure 3:**
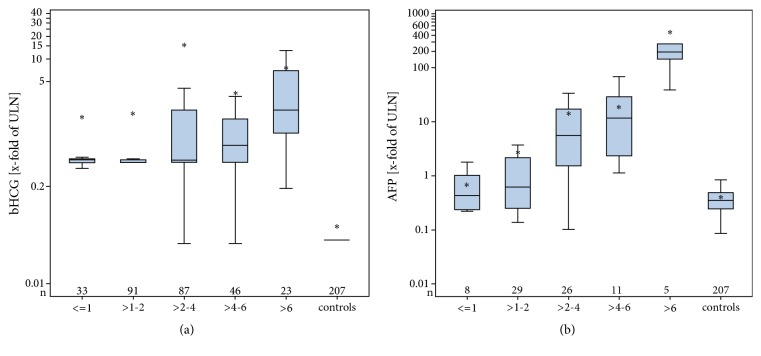
(a) Median serum levels of bHCG in categories of primary tumour size in GCT patients with clinical stage 1 (CS1). ULN: upper limit of norm (for explanations of box plot details see legend to Figure 1). (b) Median serum levels of AFP in categories of primary tumour size in nonseminoma patients with clinical stage 1 (CS1). ULN: upper limit of norm.

Likewise, the extent of marker elevation (x-fold of ULN) is significantly associated with tumour size (Table 5 and Figures 3(a) and 3(b)). AFP-levels increased significantly with increasing tumour size in nonseminoma. Levels of bHCG and LDH increased significantly with increasing tumour size in the group of all GCT patients with CS1.

Regarding age, the dichotomized evaluation revealed a significant inverse association with prevalence rates of bHCG and AFP (Table 6) in the cohort of all GCT patients. Highest rates were found in the age groups below the age of 30 years, and significantly lower rates were observed in older age groups. By contrast, LDH positivity was almost identical in all age groups. Clearly graded frequency rates in the five age groups with descending order towards older age groups were noted for the combined prevalence of both AFP and bHCG.

**Figure 4:**
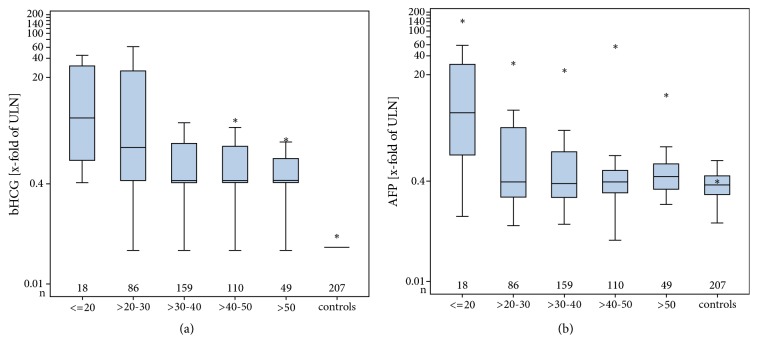
(a) Median serum levels of bHCG in age categories of all patients with GCT and controls. ULN: upper limit of norm (for explanations of box plot details see legend to Figure 1). Note significantly higher levels in younger age groups. (b) Median serum levels of AFP in age categories of all patients with GCT and controls.

The extent of elevation of AFP and bHCG was likewise significantly associated with age, showing the highest levels in patients younger than 20 years and lowest levels in those aged 50 years or more (Table 6(b), Figures 4(a) and 4(b)). Noteworthy, LDH levels did not show any association with age.

### 3.3. Marker Elevation Rates in Response to Treatment and in Relapsing Patients

In repeat measurements of patients with CS1 before and after orchiectomy, the dichotomized evaluation showed significant reduction of elevation rates of all three markers after surgery (Table 7). At completion of adjuvant therapy elevation rates of bHCG and AFP further decreased to 1-1.3% while LDH elevation rates remained in the range of 10%, in both seminoma and nonseminoma. Likewise, with regard to extent of marker elevation, the median values dropped significantly from the time of diagnosis to the time of completion of treatment (data not shown).

In metastasized disease (CS2-3), orchiectomy resulted in a small but significant decrease of frequencies of elevated levels of bHCG and LDH but not of AFP in all GCT patients and in the nonseminoma subgroup (Table 8(a)). The dichotomized evaluation further disclosed a significant decrease of the prevalence rates of all three markers after one course of chemotherapy in all GCT and in nonseminomas. At completion of therapy, only very few cases (2.8% - 4.5%) still had elevated levels of bHCG and AFP. But notably, 30.5% of all patients (34.1% of nonseminomas) with metastases at first diagnosis had persisting elevations of LDH at completion of therapy.

**Figure 5:**
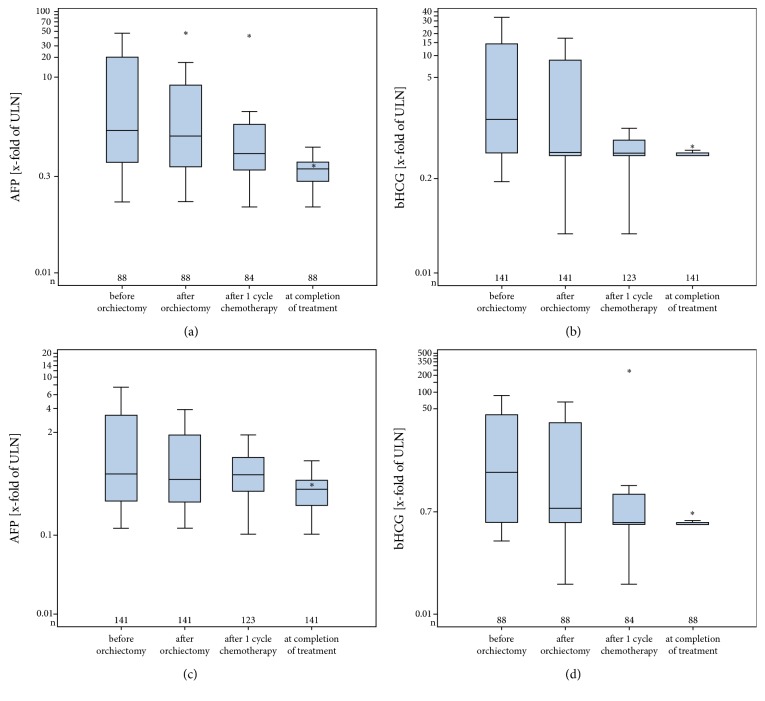
(a) Median serum levels of AFP at various points of time during treatment of all GCT patients with systemic disease (>CS1). ULN: upper limit of norm. Note decrease of levels during the course of treatment (for explanations of box plot details see legend to Figure 1). (b) Median serum levels of bHCG at various points of time during treatment of all GCT patients with systemic disease (>CS1). (c) Median serum levels of AFP at various points of time during treatment of nonseminoma patients with systemic disease (>CS1). (d) Median serum levels of bHCG at various points of time during treatment of nonseminoma patients with systemic disease (>CS1).

Likewise, median measured serum levels of all three markers decreased significantly from diagnosis to completion of therapy in the entire GCT group (Table 8(b), Figures 5(a) and 5(b)). The very clear-cut drop of marker levels upon treatment of nonseminoma is illustrated in Figures 5(c) and 5(d).

Table 9(a) shows the dichotomized evaluation of marker prevalence in patients with and without relapses. In 48.1% of the relapsing patients at least one of the 3 markers was elevated. At the time of first diagnosis there were no significant differences of marker elevation rates between the patients faring without relapse and those destined to experience relapse in the later course. At the time of relapse, the frequencies of marker elevations were not significantly different from those observed in the same patients at the time of diagnosis.

The median marker levels of the nonseminoma patients going to have relapse were not significantly different from those faring without relapse at the time of first diagnosis (Table 9(b)). Likewise, the median marker levels of the relapsing patients at the time of diagnosis were not significantly different from the levels found at relapse.

In 27 of the 32 relapsing patients serial marker levels were available (Table 10). The pattern of marker elevations noted at diagnosis was found converted in 13 patients at the time of relapse. Looking only to elevations of bHCG and AFP, 5 patients lost positivity of these markers at relapse while 3 patients without bHCG elevation at diagnosis presented with bHCG elevation at relapse.

## 4. Discussion

The present investigation is the most comprehensive analysis of serum tumour marker elevation rates in germ cell tumours to date. The results are somehow unique because the study features the findings in unselected patients managed in a primary care setting during the first two decades of this century. There are four central results. (1) The elevation rates of each of the markers AFP, bHCG, and LDH in the entire GCT population are clearly less than 50%, and even the elevation of any of the three markers is encountered in less than 60% of patients. (2) The marker elevation rate is significantly associated with histological subgroups, clinical stages, local pathological (pT) stages, with size of the primary tumour, and younger age. (3) The well-known association of marker levels with response to treatment was confirmed, but notably, LDH remained elevated despite cure in more than 30% of patients. (4) At relapse, nearly one-half of the patients had elevated serum makers; however, the pattern of markers changed in almost half of these patients.

### 4.1. Marker Elevation Rates in Histologic Subgroups

The biosynthesis of AFP is confined to yolk sac tumour components of nonseminomatous GCTs while bHCG is produced in syncytiotrophoblastic-like cells occurring in both nonseminomas and seminomas [8, 38]. LDH is unspecific and secreted by all kinds of GCT cells [39] and a number of other cancers [40, 41]. Because of the histologic variability of GCTs, not all of the patients do actually have measurable serum levels of these markers [42]. Accordingly, most of the studies on markers in GCT report frequencies of marker elevations separately for nonseminomas and pure seminomas, respectively [27, 43].

Regarding the entire cohort of GCT patients, we observed elevations of bHCG, LDH, and AFP in 37.9%, 32.9%, and 25.6%, respectively. Elevation of either AFP or bHCG was found in 47.6% and elevation of any of the three markers in 59.5%. These results closely mirror the results of a recent German study on potential new markers [44]. Only few other studies report frequencies of marker elevations relating to the entire GCT population. Lippert and Javadpour observed rates of 65%, 63%, and 59% for bHCG, AFP, and LDH, respectively, and a rate of 82% for the combination of all three markers [45]. These rates are much higher than those found in our series. This difference most probably relates to the higher proportion of nonseminomas in that series and the higher proportion of cases with advanced disease. Our results are almost identical with the findings of a Spanish study [35] that represented a population-based patient series like ours. Different results were observed in a national survey in the US (n=1113) where only 16.5% of GCT patients were found to have AFP elevations. In the same study, bHCG elevations were found in 33.5% and a somewhat higher rate of LDH elevations of 41.3% [46]. Conversely, an AFP elevation rate of 26.2% of all GCT patients was reported from Denmark in a population of 603 patients while only 19% of GCT patients had bHCG elevations in that study [18]. Also in discordance with our results are the findings of a German series of 145 GCT patients where AFP elevations were noted in 35.7%, while bHCG and LDH were within the range of the present report [26]. In a study on 1100 Japanese patients with GCT, elevations of bHCG, AFP, and LDH were reported in 57.2%, 25.7%, and 52.7%, respectively [47]. The low rate of AFP elevation rate mirrors the findings of our series, but the comparatively high elevation rates of bHG and LDH are at variance. The differences among these studies probably relate to dissimilar proportions of histologic subtypes and to different stage distributions in the corresponding patient samples. Putatively, ethnic and geographic differences of the various populations studied can also explain the variation of marker elevation rates among the reported studies. Accordingly, in a recent cohort of Chinese GCT patients, prevalence rates of elevated bHCG levels and AFP-levels were reported to be as high as 70% and 48%, respectively [48]. Yet, these data show that the over-all frequency of tumour marker elevation is less than 50% regarding all GCT patients at least in those of Caucasian descent and even the elevation rate of any of the markers is encountered in less than 60%. This lack of marker positivity in roughly one-half of the GCT patients fueled the search for novel markers in the past, and actually, the recently identified microRNA-371a-3p and allied markers appear to constitute a promising novel serum tumour marker [28, 44, 49].

Regarding tumour marker elevation rates in nonseminomas, AFP had the highest rate with 60.1% of our cases. BHCG and LDH were elevated in 53% and 38.7%, respectively. Either bHCG or AFP was elevated in 73.8% and any marker elevation was observed in 81.5%. These data mirror the pivotal study of Lippert and Javadpour from 1981 [45]. Our data are also in line with the results of a large Spanish study where AFP was found to be the most prevalent marker in nonseminoma with 70% while bHCG was elevated in 53% [25]. There is a marked paucity of systematic investigations of tumour markers in GCT reported in this century [50].  Table 11 summarizes twelve studies of reasonable size that provide data on marker measurements in nonseminoma patients [17–23, 25, 26, 28, 30]. Elevated AFP-levels were observed in 52 to 72%. BHCG elevation rates are clearly lower than those of AFP with rates of 30 to 63%. LDH was specified in only two studies where rates 36% to 59% were found in nonseminoma [28, 45].

In seminoma, we found bHCG elevations in 28% of patients.  Table 12 summarizes results from other investigations [17, 18, 23, 25, 26, 28–35]. BHCG elevation rates range from 7% to 35% of seminoma cases. The LDH elevation rate of 29% in our patients accords with the rate of 34% reported by Weissbach [34] but a Norwegian study reported a higher rate of 46% [32]. Overall, patients with seminoma have significantly lower elevation rates of all tumour markers than patients with nonseminoma. Accordingly, the median serum level of bHCG was significantly lower in seminoma than in nonseminoma because many of the seminoma patients have only slightly elevated serum levels of this marker (Figures 1(a) and 1(b)). While the proportion of patients having elevated LDH levels is significantly higher in nonseminoma (38.7%) than in seminoma (29.1%), the median measured LDH serum levels of the two subgroups are not different from each other. The data accumulated here suggest that about 30% of seminoma patients have elevated levels of bHCG or LDH. Thus, these markers are helpful only for a minority of patients and from a clinical point of view a more sensitive marker would be desirable.

### 4.2. Association of Tumour Markers with Clinical Characteristics

The elevation rates of all of the three markers were significantly associated with clinical stages in the cohort of all GCT patients. Median measured serum levels of the three markers are likewise associated with clinical stages showing the lowest levels in CS1 and the highest in CS3. Noteworthy, in the cohort of nonseminoma patients, elevation rates of bHCG and LDH are associated with stages but AFP is not. In nonseminoma, CS1 cases and those with higher stages (>CS1) have almost identical proportions of cases with elevated AFP of 62.5% and 58%, respectively. These findings are in accordance with data reported by Kausitz et al. who observed almost identical elevation rates of AFP in nonseminoma patients with CS1 and in those with higher stages (>CS1) [22]. Likewise, the median serum levels of AFP are not significantly different among clinical stages in our series. Our findings are in conflict with an early report of Skinner and Scardino who noted higher rates of elevations of both bHCG and AFP in CS3 than in CS2 cases but found only rates of 7% and 9% in CS1 cases with positivity for bHCG and AFP [51]. However, in that series only postorchiectomy measurements of the markers were considered. Our findings are also in contrast to the report of the International Germ Cell Cancer Consensus Group (IGCCCG) where the three different prognostic groups of GCT patients had clearly graded median serum levels of tumor markers [52]. The reason why the median AFP-levels of our nonseminoma patients were not different among the clinical stages is unclear. However, a chance finding must be considered because our series comprised of a much lower number of metastasized patients (n=141) than the IGCCCG meta-analysis (>5000).

Generally, higher rates of marker elevation in advanced clinical stages had first been noted by Lippert and Javadpour in 1980 [45] and this association of marker elevation rates with clinical staging was confirmed by many other investigators [17, 18, 20, 21, 24, 30, 51, 53, 54]. The most probable biological reason for this association is the increase of marker-producing tumour cells with increasing clinical stages.

Regarding pT-stages, elevation rates of bHCG and LDH are significantly higher in GCT patients with advanced pT-stages (>pT1) than in those with organ confined tumours (pT1), but notably the rates of AFP are not. Likewise, median measured serum levels of bHCG and LDH were associated with pT-staging but again, AFP-levels were not. The associations of bHCG and LDH with pT-staging are in accordance with biological expectations because, usually, pT1 stage denotes a tumour confined to the testicular compartment, the tumour has not yet invaded the vascular drainage system and many of these tumours are rather small. Thus, the frequencies of tumour marker elevations as well as the median measured marker levels in peripheral serum are expected to be lower in this group than in advanced local tumour stages where the malignancy has gained access to surrounding structures. The reason why AFP is not associated with pT-staging in the whole group of GCT patients is probably the lack of production of this marker in the large subgroup of seminoma. No previous study has so far reported the association of tumour marker elevation rates and median marker levels with pT-staging.

In line with the correlation of pT-staging with the frequencies of marker elevations is our finding of increasing marker elevation rates with increasing tumour sizes. In the cohort of GCT patients with CS1, the association is significant for bHCG and LDH, only, whereas in the nonseminomas, the elevation rates of all of the three markers are significantly associated with tumour size. Association of the extent of marker level elevation with tumour size was found for AFP in the nonseminoma group and for bHCG and LDH in the entire GCT group (Figures 3(a) and 3(b)). These associations mirror the association of marker elevation rates with clinical stages and with pT-staging and are likewise most probably caused by the higher numbers of marker-producing cells in the larger primary tumours. Significantly higher rates of elevated LDH levels had previously been noted in patients with tumours larger than 6 cm than in those with smaller primaries [55]. A significant correlation between primary tumour size and the extent of AFP and bHCG elevation was reported in a small Spanish study in 1984 [56], but no further systematic evaluations of this issue have been reported to date.

A novel finding is the inverse association of elevation rates of AFP and bHCG with age in the entire group of GCT patients; i.e., young patients have higher rates of elevated tumour markers than the older ones. This result contrasts with the reported higher rates of bHCG elevations in the older age groups of the healthy male population [57]. But obviously, in elderly healthy males only mildly elevated levels will be encountered and these elevations are always associated with increased levels of luteinizing hormone (LH), indicating chemical cross reactions with that hormone in the presence of late onset hypogonadism. LDH is not associated with age. In the nonseminoma group, only AFP is associated with age. Likewise, the median serum levels of AFP and bHCG are associated with age in the entire GCT cohort (Figures 4(a) and 4(b)). In nonseminoma, only AFP-levels are significantly associated with age. Again, LDH is not associated with age regarding the median measured serum levels. The biological background for this finding is probably the high incidence of AFP-producing yolk sac tumours and bHCG producing choriocarcinomas in the younger patients while the nonsecreting seminomas predominate in the older age groups. LDH is synthesized by all histological subgroups of GCT and therefore no age predisposition is found for this marker.

### 4.3. Marker Elevation Rates in Response to Treatment and at Relapse

A premier role of serum tumor markers is to monitor the course of clinical management and to early herald success or treatment failure [1, 4, 11, 58]. Accordingly, marker decline indicating response to therapy has been documented in the very early reports after the upcoming of the three markers [17, 42, 59–70]. In accordance with these reports, we observed significant decreases of elevation rates of all three markers after orchiectomy in CS1 patients with further dropping to rates around 1% after completion of treatment (i.e., after adjuvant therapy) regarding bHCG and AFP. Notably, the LDH elevation rate did not drop to that extent. This marker kept having positivity rates of around 10% after completion of treatment in both seminoma and nonseminoma. In systemic disease (CS>1) a very similar decrease of elevation rates is observed. The rates of bHCG and AFP revealed decreases after each step of treatment to finish with rates around 3% at completion of treatment. By contrast, the frequency of LDH elevation showed only some decrease in response to treatment but the serum levels remained elevated in around 30% after entering complete remission in both seminoma and nonseminoma. Such false-positive elevations of LDH in tumour-free patients have been noted earlier [32]. Median measured marker levels showed significant decreases in response to treatment with respect to all three markers (Figures 4(a) and 4(b)). One would have expected zero elevation rates of the markers after successful treatment because at this time-point no cancer cells are expected to exist anymore and produce any marker substances. The persisting very low rates of AFP and bHCG elevation after treatment of GCT may be explained by some few treatment failures and or by the well-known low rate of false positive elevation of AFP and bHCG in men without active GCT [71]. Overall, our findings plainly confirm the value of AFP and bHCG for monitoring treatment of GCT in those cases where the markers are elevated. As noted earlier, LDH is much more unspecific for GCT than AFP and bHCG because this enzyme is released from cells of many organs of the body at apoptosis. Notably, as many as 8.2% of young men of our control group without malignancy had elevations of LDH. Accordingly, the persisting elevation rates of 10 – 34% after GCT treatment in the absence of disease are a striking finding [32]. One reason for the persisting elevation of LDH in a substantial number of patients could be the known quite long half-life of this enzyme. In contrast to AFP and bHCG, the LDH decay is rather long with 1 – 3 weeks and, moreover, it may vary with clinical staging [72]. Thus, the persisting high rates of LDH elevation after treatment may relate to prolonged decay but may also indicate the low specificity of this marker for GCT. In all, the usefulness of LDH in clinical management of GCT must obviously be questioned as already stated by other authorities [73–75].

Increasing marker levels during follow-up may herald recurrent disease, as demonstrated by many authors [17, 18, 20, 22, 42, 50, 76–78] although its use in seminoma had been questioned [34, 79, 80]. In our study, there were slightly lower frequencies of marker elevation at the time of relapse than at the time of first diagnosis but these differences were not significant, statistically. This observation is in accordance with previous reports [22, 81, 82]. It is of clinical relevance that among the 27 patients developing relapse almost half of whom (13 of 27) experienced a change of the individual marker pattern. This finding is at variance with a Spanish investigation that reported only one-third of relapsing patients with changed marker patterns [81] and a Dutch study with only 3 of 17 relapsing patients developing a change of the marker pattern [76]. The possible pattern change upon relapse underscores the need for measuring the serum levels of all markers during follow-up.

Limitations of our study relate to a possible selection bias because of the retrospective design. Some of the various subgroups tested involve only small patient numbers allowing for statistical chance results in some calculations. On the other hand, strengths of the study involve the large number of cases examined, the completeness of data sets in more than 90% of cases, and the exceptional quantity of detailed information collected.

### 4.4. Conclusions

AFP and bHCG are valuable tools for the clinical management of GCTs but LDH is clearly of limited value. A major shortcoming of these markers is the low frequency of elevated serum levels in less than 50% in the entire cohort of GCT patients. The frequencies of elevated marker levels rates are significantly associated with histology, higher clinical stages, and other clinical factors such as younger age, higher pT-stages, and primary tumour size. Serum levels of AFP and bHCG decrease in response to therapy while LDH continues to be expressed in around 30% of patients after entering complete remission. At relapse only one-quarter of patients have elevated levels of AFP or bHCG, and importantly, the individual marker pattern changed in comparison to that at first diagnosis in almost one half of these patients.

Current guidelines recommend the measurement of the three serum markers discussed herein as one cornerstone of the clinical management of testicular cancer [6, 11]. However, a promising new generation of serum biomarkers of GCTs such as serum levels of microRNAs is presently waiting for clinical implementation. As recently documented, these new epigenetic markers particularly serum levels of microRNA-371a-3p outperform the classical markers by far with a sensitivity of 90.1% and a specificity of 94.0% [28, 83]. Due to its exceptionally high sensitivity, this marker—in contrast to the classical markers—may aid in establishing the primary diagnosis of GCT [84]. As the microRNA-371a-3p is also elevated in 82% of patients with recurrent disease, this marker may also be helpful for the early detection of relapses [85]. Clearly, the traditional markers of GCT are currently indispensable for the clinical management of GCT despite their limitations. But, serum levels of microRNA-371a-3p represent a possibly more powerful tool and the future will show if bHCG, AFP, and LDH will be supplemented or even replaced by the new generation of markers.

## Figures and Tables

**Table 1:** Frequencies of elevated marker levels in the entire GCT cohort at diagnosis.

		bHCG elevated	AFP elevated	LDH elevated	bHCG and AFP elevated	bHCG and LDH elevated	AFP and LDH elevated	bHCG, AFP and LDH elevated	bHCG or AFP elevated	bHCG, AFP or LDH elevated
	N	n (%)	n (%)	n (%)	n (%)	n (%)	n (%)	n (%)	n (%)	n (%)
		95% CI [%]	95% CI [%]	95% CI [%]	95% CI [%]	95% CI [%]	95% CI [%]	95% CI [%]	95% CI [%]	95% CI [%]
*Entire GCT cohort *	422	160 (37.9%)	108 (25.6%)	139 (32.9%)	67 (15.9%)	73 (17.3%)	46 (10.9%)	30 (7.1%)	201 (47.6%)	251 (59.5%)
		33.3 - 42.8	21.6 - 30.1	28.5 - 37.7	12.6 - 19.8	13.9 - 21.3	8.2 - 14.4	4.9 - 10.1	42.8 - 52.5	54.6 - 64.2
*Pathological stage *										
pT1	222	59 (26.6%)	54 (24.3%)	48 (21.6%)	28 (12.6%)	14 (6.3%)	18 (8.1%)	9 (4.1%)	85 (38.3%)	110 (49.5%)
		21.0 - 33.0	18.9 - 30.6	16.5 - 27.7	8.7 - 17.9	3.6 - 10.6	5.0 - 12.7	2.0 - 7.8	31.9 - 45.1	42.8 - 56.3
>pT1	200	101 (50.5%)	54 (27.0%)	91 (45.5%)	39 (19.5%)	59 (29.5%)	28 (14.0%)	21 (10.5%)	116 (58.0%)	141 (70.5%)
		43.4 - 57.6	21.1 - 33.8	38.5 - 52.7	14.4 - 25.8	23.4 - 36.4	9.7 - 19.8	6.8 - 15.8	50.8 - 64.9	63.6 - 76.6
p-value^a^		<.0001	0.5294	<.0001	0.0532	<.0001	0.0503	0.0097	<.0001	<.0001
*Clinical stage *										
CS1	281	86 (30.6%)	54 (19.2%)	64 (22.8%)	31 (11.0%)	25 (8.9%)	15 (5.3%)	8 (2.8%)	109 (38.8%)	141 (50.2%)
		25.3 - 36.4	14.9 - 24.4	18.1 - 28.2	7.7 - 15.4	6.0 - 13.0	3.1 - 8.8	1.3 - 5.7	33.1 - 44.8	44.2 - 56.2
CS2a,b	95	44 (46.3%)	33 (34.7%)	43 (45.3%)	22 (23.2%)	25 (26.3%)	18 (18.9%)	13 (13.7%)	55 (57.9%)	68 (71.6%)
		36.1 - 56.8	25.5 - 45.3	35.1 - 55.8	15.4 - 33.2	18.1 - 36.5	11.9 - 28.6	7.8 - 22.6	47.3 - 67.8	61.3 - 80.1
CS2c	17	9 (52.9%)	5 (29.4%)	11 (64.7%)	2 (11.8%)	7 (41.2%)	2 (11.8%)	1 (5.9%)	12 (70.6%)	15 (88.2%)
		28.5 - 76.1	11.4 - 56.0	38.6 - 84.7	2.1 - 37.7	19.4 - 66.5	2.1 - 37.7	0.3 - 30.8	44.0 - 88.6	62.3 - 97.9
CS3	29	21 (72.4%)	16 (55.2%)	21 (72.4%)	12 (41.4%)	16 (55.2%)	11 (37.9%)	8 (27.6%)	25 (86.2%)	27 (93.1%)
		52.5 - 86.6	36.0 - 73.0	52.5 - 86.6	24.1 - 60.9	36.0 - 73.0	21.3 - 57.6	13.4 - 47.5	67.4 - 95.5	75.8 - 98.8
p-value^a^		<.0001	<.0001	<.0001	<.0001	<.0001	<.0001	<.0001	<.0001	<.0001
*Localisation *										
Right side	201	67 (33.3%)	47 (23.4%)	64 (31.8%)	27 (13.4%)	30 (14.9%)	21 (10.4%)	14 (7.0%)	87 (43.3%)	114 (56.7%)
		27.0 - 40.4	17.8 - 30.0	25.6 - 38.8	9.2 - 19.1	10.4 - 20.8	6.7 - 15.7	4.0 - 11.6	36.4 - 50.4	49.6 - 63.6
Left side	200	85 (42.5%)	56 (28.0%)	69 (34.5%)	37 (18.5%)	38 (19.0%)	22 (11.0%)	14 (7.0%)	104 (52.0%)	127 (63.5%)
		35.6 - 49.7	22.0 - 34.9	28.0 - 41.6	13.5 - 24.7	13.9 - 25.3	7.2 - 16.4	4.0 - 11.7	44.9 - 59.1	56.4 - 70.1
p-value^a^		0.0585	0.2900	0.5717	0.1660	0.2770	0.8582	0.9891	0.0806	0.1654
*controls *	208	0 (0.0%)	3 (1.4%)	17 (8.2%)	0 (0.0%)	0 (0.0%)	0 (0.0%)	0 (0.0%)	3 (1.4%)	20 (9.6%)
		0.0 - 3.1	0.4 - 4.5	5.0 - 13.0	0.0 - 3.1	0.0 - 3.1	0.0 - 3.1	0.0 - 3.1	0.4 - 4.5	6.1 - 14.7

^A^   chi square test for comparisons.

**Table 2:** Frequencies of elevated marker levels in seminoma and nonseminoma at the time of diagnosis.

		bHCG elevated	AFP elevated	LDH elevated	bHCG and AFP elevated	bHCG and LDH elevated	AFP and LDH elevated	bHCG, AFP and LDH elevated	bHCG or AFP elevated	bHCG, AFP or LDH elevated
	N	n (%)	n (%)	n (%)	n (%)	n (%)	n (%)	n (%)	n (%)	n (%)
		95% CI [%]	95% CI [%]	95% CI [%]	95% CI [%]	95% CI [%]	95% CI [%]	95% CI [%]	95% CI [%]	95% CI [%]
*Seminoma*	254	71 (28.0%)	7 (2.8%)	74 (29.1%)	1 (0.4%)	34 (13.4%)	4 (1.6%)	1 (0.4%)	77 (30.3%)	114 (44.9%)
		22.6 - 34.0	1.2 - 5.8	23.7 - 35.2	0.0 - 2.5	9.6 - 18.3	0.5 - 4.3	0.0 - 2.5	24.8 - 36.4	38.7 - 51.2
*Nonseminoma*	168	89 (53.0%)	101 (60.1%)	65 (38.7%)	66 (39.3%)	39 (23.2%)	42 (25.0%)	29 (17.3%)	124 (73.8%)	137 (81.5%)
		45.2 - 60.7	52.3 - 67.5	31.4 - 46.5	31.9 - 47.1	17.2 - 30.5	18.8 - 32.4	12.0 - 24.0	66.4 - 80.1	74.7 - 86.9
p-value^a^		<.0001	<.0001	0.0367	<.0001	0.0082	<.0001	<.0001	<.0001	<.0001
*CS1 vs. >CS1 (Seminoma) *										
CS1	201	49 (24.4%)	4 (2.0%)	46 (22.9%)	0 (0.0%)	17 (8.5%)	3 (1.5%)	0 (0.0%)	53 (26.4%)	79 (39.3%)
		18.7 - 31.0	0.6 - 5.3	17.4 - 29.4	0.0 - 2.3	5.2 - 13.4	0.4 - 4.7	0.0 - 2.3	20.5 - 33.1	32.6 - 46.4
>CS1	53	22 (41.5%)	3 (5.7%)	28 (52.8%)	1 (1.9%)	17 (32.1%)	1 (1.9%)	1 (1.9%)	24 (45.3%)	35 (66.0%)
		28.4 - 55.8	1.5 - 16.6	38.8 - 66.5	0.1 - 11.4	20.3 - 46.4	0.1 - 11.4	0.1 - 11.4	31.8 - 59.4	51.6 - 78.1
p-value^a^		0.0134	0.1465	<.0001	0.0510	<.0001	0.8375	0.0510	0.0077	0.0005
*CS1 vs. >CS1 (Nonseminoma) *										
CS1	80	37 (46.3%)	50 (62.5%)	18 (22.5%)	31 (38.8%)	8 (10.0%)	12 (15.0%)	8 (10.0%)	56 (70.0%)	62 (77.5%)
		35.2 - 57.7	50.9 - 72.9	14.2 - 33.5	28.3 - 50.3	4.7 - 19.3	8.3 - 25.1	4.7 - 19.3	58.6 - 79.5	66.5 - 85.8
>CS1	88	52 (59.1%)	51 (58.0%)	47 (53.4%)	35 (39.8%)	31 (35.2%)	30 (34.1%)	21 (23.9%)	68 (77.3%)	75 (85.2%)
		48.1 - 69.3	47.0 - 68.2	42.5 - 64.0	29.7 - 50.8	25.5 - 46.2	24.5 - 45.1	15.7 - 34.4	66.9 - 85.2	75.7 - 91.6
p-value^a^		0.0958	0.5479	<.0001	0.8922	0.0001	0.0049	0.0193	0.2843	0.1972
*Nonseminoma with teratoma *										
No teratoma	88	40 (45.5%)	43 (48.9%)	36 (40.9%)	27 (30.7%)	17 (19.3%)	20 (22.7%)	11 (12.5%)	56 (63.6%)	66 (75.0%)
		34.9 - 56.4	38.1 - 59.7	30.7 - 51.9	21.5 - 41.5	12.0 - 29.4	14.8 - 33.1	6.7 - 21.7	52.6 - 73.4	64.4 - 83.4
With teratoma	80	49 (61.3%)	58 (72.5%)	29 (36.3%)	39 (48.8%)	22 (27.5%)	22 (27.5%)	18 (22.5%)	68 (85.0%)	71 (88.8%)
		49.7 - 71.7	61.2 - 81.6	26.0 - 47.8	37.5 - 60.1	18.4 - 38.8	18.4 - 38.8	14.2 - 33.5	74.9 - 91.7	79.2 - 94.4
p-value^a^		0.0405	0.0018	0.5783	0.0166	0.1933	0.4464	0.0798	0.0017	0.0218

^a^  Chi square test for comparisons of subgroups.

**Table 3:** Extent of marker elevation at diagnosis.

		bHCG [x-fold of ULN]			AFP [x-fold of ULN]			LDH [x-fold of ULN]		
	n (%)	Min	Median (Q1 - Q3)	Max	Min	Median (Q1 - Q3)	Max	Min	Median (Q1 - Q3)	Max
*All GCT patients *	422 (100.0%)	0.03	0.46 (0.41 - 3.12)	749063.67	0.05	0.40 (0.24 - 1.04)	5906.58	0.01	0.89 (0.76 - 1.10)	17.00
*pT-stage*										
pT1	222 (52.6%)	0.03	0.45 (0.41 - 1.08)	749063.67	0.05	0.38 (0.23 - 0.86)	5906.58	0.01	0.83 (0.72 - 0.96)	17.00
>pT1	200 (47.4%)	0.03	1.03 (0.45 - 4.93)	130974.14	0.08	0.48 (0.25 - 1.20)	1630.46	0.03	0.97 (0.82 - 1.24)	9.32
p-value^a^			<.0001			0.1126			<.0001	
*Histology *										
Seminoma	254 (60.2%)	0.03	0.45 (0.41 - 1.16)	86.23	0.05	0.30 (0.22 - 0.47)	2.81	0.01	0.88 (0.75 - 1.06)	8.40
Nonseminoma	168 (39.8%)	0.03	1.35 (0.45 - 18.65)	749063.67	0.10	2.02 (0.49 - 13.02)	5906.58	0.55	0.90 (0.78 - 1.12)	17.00
p-value^a^			<.0001			<.0001			0.0963	
*Clinical stage *										
CS1	281 (66.6%)	0.03	0.45 (0.41 - 1.31)	560.26	0.05	0.36 (0.23 - 0.64)	1630.46	0.01	0.83 (0.72 - 0.98)	5.41
CS2a,b	95 (22.5%)	0.18	0.73 (0.45 - 12.94)	19427.83	0.13	0.50 (0.25 - 2.34)	195.55	0.03	0.98 (0.84 - 1.13)	8.40
CS2c	17 (4.0%)	0.22	1.20 (0.41 - 30.19)	28667.79	0.17	0.67 (0.45 - 1.93)	1596.20	0.59	1.34 (0.95 - 2.48)	3.80
CS3	29 (6.9%)	0.34	6.78 (0.79 - 70.86)	749063.67	0.12	1.35 (0.42 - 51.31)	5906.58	0.82	1.28 (0.96 - 1.68)	17.00
p-value^a^			<.0001			<.0001			<.0001	
*CS1 vs. >CS1 *										
CS1	281 (66.6%)	0.03	0.45 (0.41 - 1.31)	560.26	0.05	0.36 (0.23 - 0.64)	1630.46	0.01	0.83 (0.72 - 0.98)	5.41
>CS1	141 (33.4%)	0.18	1.31 (0.45 - 14.38)	749063.67	0.12	0.59 (0.27 - 3.29)	5906.58	0.03	1.04 (0.88 - 1.42)	17.00
p-value^a^			<.0001			<.0001			<.0001	
*CS1 vs. >CS1 (Seminoma) *										
CS1	201 (79.1%)	0.03	0.45 (0.41 - 0.94)	47.73	0.05	0.30 (0.22 - 0.46)	2.12	0.01	0.83 (0.73 - 0.98)	5.41
>CS1	53 (20.9%)	0.18	0.69 (0.45 - 3.37)	86.23	0.13	0.28 (0.23 - 0.50)	2.81	0.03	1.03 (0.88 - 1.58)	8.40
p-value^a^			0.0001			0.3529			<.0001	
*CS1 vs. >CS1 (Nonseminoma) *										
CS1	80 (47.6%)	0.03	0.72 (0.43 - 4.86)	560.26	0.10	2.27 (0.48 - 12.02)	1630.46	0.55	0.81 (0.72 - 0.98)	2.71
>CS1	88 (52.4%)	0.21	3.59 (0.45 - 39.12)	749063.67	0.12	1.51 (0.49 - 20.16)	5906.58	0.57	1.04 (0.87 - 1.31)	17.00
p-value^a^			0.0041			0.9671			<.0001	

^a^  Mann-Whitney U test for comparisons of subgroups; Min minimum value, Max maximum value, ULN upper limit of norm, Q1: first quartile, Q3: third quartile.

**Table 4:** Frequencies of elevated marker levels of patients with CS1 in categories of primary tumour size.

Primary tumour size (categories)		bHCG elevated	AFP elevated	LDH elevated	bHCG and AFP elevated	bHCG and LDH elevated	AFP and LDH elevated	bHCG, AFP and LDH elevated	bHCG or AFP elevated	bHCG, AFP or LDH elevated
	N	n (%)	n (%)	n (%)	n (%)	n (%)	n (%)	n (%)	n (%)	n (%)
		95% CI [%]	95% CI [%]	95% CI [%]	95% CI [%]	95% CI [%]	95% CI [%]	95% CI [%]	95% CI [%]	95% CI [%]
*All GCT *										
<=1 cm	33	2 (6.1%)	4 (12.1%)	9 (27.3%)	1 (3.0%)	0 (0.0%)	2 (6.1%)	0 (0.0%)	5 (15.2%)	12 (36.4%)
		1.1 - 21.6	4.0 - 29.1	13.9 - 45.8	0.2 - 17.5	0.0 - 13.0	1.1 - 21.6	0.0 - 13.0	5.7 - 32.7	21.0 - 54.9
>1-2 cm	91	14 (15.4%)	10 (11.0%)	10 (11.0%)	6 (6.6%)	1 (1.1%)	0 (0.0%)	0 (0.0%)	18 (19.8%)	27 (29.7%)
		9.0 - 24.8	5.7 - 19.7	5.7 - 19.7	2.7 - 14.3	0.1 - 6.8	0.1 - 6.8	0.1 - 6.8	12.4 - 29.7	20.8 - 40.3
>2-4 cm	87	31 (35.6%)	22 (25.3%)	16 (18.4%)	15 (17.2%)	5 (5.7%)	5 (5.7%)	3 (3.4%)	38 (43.7%)	47 (54.0%)
		25.9 - 46.7	16.8 - 35.9	11.2 - 28.4	10.3 - 27.2	2.1 - 13.5	2.1 - 13.5	0.9 - 10.5	33.2 - 54.7	43.0 - 64.6
>4-6 cm	46	20 (43.5%)	12 (26.1%)	14 (30.4%)	4 (8.7%)	6 (13.0%)	3 (6.5%)	1 (2.2%)	28 (60.9%)	34 (73.9%)
		29.2 - 58.8	14.8 - 41.4	18.2 - 45.9	2.8 - 21.7	5.4 - 27.0	1.7 - 18.9	0.1 - 13.0	45.4 - 74.5	58.6 - 85.2
>6 cm	23	18 (78.3%)	5 (21.7%)	14 (60.9%)	4 (17.4%)	12 (52.2%)	4 (17.4%)	3 (13.0%)	19 (82.6%)	20 (87.0%)
		55.8 - 91.7	8.3 - 44.2	38.8 - 79.5	5.7 - 39.5	31.1 - 72.6	5.7 - 39.5	3.4 - 34.7	60.5 - 94.3	65.3 - 96.6
p-value^a^		<.0001	0.0696	<.0001	0.0664	<.0001	0.0138	0.0075	<.0001	<.0001
*Nonseminoma *										
<=1 cm	8	2 (25.0%)	2 (25.0%)	1 (12.5%)	1 (12.5%)	0 (0.0%)	0 (0.0%)	0 (0.0%)	3 (37.5%)	4 (50.0%)
		4.5 - 64.4	4.5 - 64.4	0.7 - 53.3	0.7 - 53.3	0.0 - 40.2	0.0 - 40.2	0.0 - 40.2	10.2 - 74.1	17.4 - 82.6
>1-2 cm	29	9 (31.0%)	10 (34.5%)	4 (13.8%)	6 (20.7%)	0 (0.0%)	0 (0.0%)	0 (0.0%)	13 (44.8%)	17 (58.6%)
		16.0 - 51.0	18.6 - 54.3	4.5 - 32.6	8.7 - 40.3	0.2 - 19.6	0.2 - 19.6	0.2 - 19.6	27.0 - 64.0	39.1 - 75.9
>2-4 cm	26	17 (65.4%)	21 (80.8%)	6 (23.1%)	15 (57.7%)	3 (11.5%)	5 (19.2%)	3 (11.5%)	23 (88.5%)	24 (92.3%)
		44.4 - 82.1	60.0 - 92.7	9.8 - 44.1	37.2 - 76.0	3.0 - 31.3	7.3 - 40.0	3.0 - 31.3	68.7 - 97.0	73.4 - 98.7
>4-6 cm	11	4 (36.4%)	11 (100.0%)	2 (18.2%)	4 (36.4%)	1 (9.1%)	2 (18.2%)	1 (9.1%)	11 (100.0%)	11 (100.0%)
		12.4 - 68.4	67.9 -100.0	3.2 - 52.2	12.4 - 68.4	0.5 - 42.9	3.2 - 52.2	0.5 - 42.9	67.9 -100.0	67.9 -100.0
>6 cm	5	4 (80.0%)	5 (100.0%)	4 (80.0%)	4 (80.0%)	3 (60.0%)	4 (80.0%)	3 (60.0%)	5 (100.0%)	5 (100.0%)
		29.9 - 98.9	46.3 -100.0	29.9 - 98.9	29.9 - 98.9	17.0 - 92.7	29.9 - 98.9	17.0 - 92.7	46.3 -100.0	46.3 -100.0
p-value^a^		0.0299	<.0001	0.0226	0.0075	0.0006	<.0001	0.0006	<.0001	0.0018

^a^  Chi square test for comparisons of subgroup.

**Table 5:** Extent of marker elevations (x-fold of ULN) in relation to tumour size in CS1 patients.

		bHCG [x-fold of ULN]			AFP [x-fold of ULN]			LDH [x-fold of ULN]		
Tumour size categories (cm)	n (%)	Min	Median (Q1 - Q3)	Max	Min	Median (Q1 - Q3)	Max	Min	Median (Q1 - Q3)	Max
*Nonseminoma*										
<=1	8 (10.0%)	0.34	0.52 (0.41 - 5.25)	31.69	0.22	0.43 (0.23 - 1.02)	1.79	0.64	0.77 (0.70 - 0.87)	1.22
>1-2	29 (36.3%)	0.03	0.45 (0.41 - 2.34)	49.66	0.14	0.62 (0.25 - 2.16)	22.48	0.55	0.76 (0.67 - 0.89)	1.44
>2-4	26 (32.5%)	0.03	1.97 (0.45 - 4.89)	560.26	0.10	5.58 (1.53 - 17.13)	93.72	0.59	0.81 (0.74 - 0.96)	1.25
>4-6	11 (13.8%)	0.17	0.56 (0.31 - 10.37)	64.54	1.14	11.79 (2.34 - 28.86)	68.14	0.68	0.96 (0.78 - 1.00)	1.46
>6	5 (6.3%)	0.86	2.21 (1.02 - 16.31)	83.90	38.39	194.00 (144.88 - 277.50)	1630.46	0.57	1.11 (1.06 - 1.18)	2.71
p-value^a^			0.0673			<.0001			0.0742	
*All GCT*										
<=1	33 (11.7%)	0.03	0.45 (0.41 - 0.46)	31.69	0.08	0.34 (0.22 - 0.52)	2.12	0.55	0.80 (0.71 - 1.00)	2.81
>1-2	91 (32.4%)	0.03	0.45 (0.41 - 0.45)	49.66	0.05	0.31 (0.22 - 0.59)	22.48	0.51	0.79 (0.68 - 0.89)	1.62
>2-4	87 (31.0%)	0.03	0.45 (0.41 - 2.08)	560.26	0.08	0.36 (0.23 - 1.07)	93.72	0.38	0.81 (0.74 - 0.92)	1.28
>4-6	46 (16.4%)	0.03	0.70 (0.41 - 1.58)	64.54	0.11	0.42 (0.22 - 1.14)	68.14	0.67	0.90 (0.77 - 1.05)	1.81
>6	23 (8.2%)	0.19	2.07 (1.02 - 6.99)	83.90	0.17	0.34 (0.24 - 0.49)	1630.46	0.01	1.11 (0.94 - 1.50)	5.41
p-value^a^			<.0001			0.4388			<.0001	

^a^  Mann-Whitney U Test for comparisons of subgroups.

**Table:** (a) Frequencies of elevated marker levels rates in age groups

		bHCG elevated	AFP elevated	LDH elevated	bHCG and AFP elevated	bHCG and LDH elevated	AFP and LDH elevated	bHCG, AFP and LDH elevated	bHCG or AFP elevated	bHCG, AFP or LDH elevated
Age categories (years)	N	n (%)	n (%)	n (%)	n (%)	n (%)	n (%)	n (%)	n (%)	n (%)
		95% CI [%]	95% CI [%]	95% CI [%]	95% CI [%]	95% CI [%]	95% CI [%]	95% CI [%]	95% CI [%]	95% CI [%]
*All GCT *										
<=20	18	13 (72.2%)	14 (77.8%)	9 (50.0%)	12 (66.7%)	7 (38.9%)	8 (44.4%)	7 (38.9%)	15 (83.3%)	16 (88.9%)
		46.4 - 89.3	51.9 - 92.6	26.8 - 73.2	41.2 - 85.6	18.3 - 63.9	22.4 - 68.7	18.3 - 63.9	57.7 - 95.6	63.9 - 98.1
>20-30	86	47 (54.7%)	29 (33.7%)	25 (29.1%)	22 (25.6%)	14 (16.3%)	8 (9.3%)	6 (7.0%)	54 (62.8%)	63 (73.3%)
		43.6 - 65.3	24.1 - 44.8	20.0 - 40.0	17.1 - 36.3	9.5 - 26.1	4.4 - 18.0	2.9 - 15.1	51.6 - 72.8	62.4 - 82.0
>30-40	159	51 (32.1%)	44 (27.7%)	53 (33.3%)	22 (13.8%)	27 (17.0%)	20 (12.6%)	13 (8.2%)	73 (45.9%)	92 (57.9%)
		25.0 - 40.0	21.0 - 35.4	26.2 - 41.3	9.1 - 20.4	11.7 - 23.9	8.0 - 19.0	4.6 - 13.9	38.1 - 54.0	49.8 - 65.6
>40-50	110	36 (32.7%)	12 (10.9%)	38 (34.5%)	7 (6.4%)	19 (17.3%)	7 (6.4%)	3 (2.7%)	41 (37.3%)	56 (50.9%)
		24.3 - 42.4	6.0 - 18.6	25.9 - 44.3	2.8 - 13.1	11.0 - 25.9	2.8 - 13.1	0.7 - 8.4	28.4 - 47.1	41.3 - 60.5
>50	49	13 (26.5%)	9 (18.4%)	14 (28.6%)	4 (8.2%)	6 (12.2%)	3 (6.1%)	1 (2.0%)	18 (36.7%)	24 (49.0%)
		15.4 - 41.3	9.2 - 32.5	17.0 - 43.5	2.6 - 20.5	5.1 - 25.5	1.6 - 17.9	0.1 - 12.2	23.8 - 51.7	34.6 - 63.5
p-value^a^		<.0001	<.0001	0.4679	<.0001	0.1468	<.0001	<.0001	<.0001	0.0008
*nonseminoma *										
<=20	17	12 (70.6%)	14 (82.4%)	9 (52.9%)	12 (70.6%)	7 (41.2%)	8 (47.1%)	7 (41.2%)	14 (82.4%)	15 (88.2%)
		44.0 - 88.6	55.8 - 95.3	28.5 - 76.1	44.0 - 88.6	19.4 - 66.5	23.9 - 71.5	19.4 - 66.5	55.8 - 95.3	62.3 - 97.9
>20-30	63	36 (57.1%)	29 (46.0%)	19 (30.2%)	22 (34.9%)	10 (15.9%)	8 (12.7%)	6 (9.5%)	43 (68.3%)	50 (79.4%)
		44.1 - 69.3	33.6 - 59.0	19.6 - 43.2	23.6 - 48.1	8.3 - 27.7	6.0 - 24.0	3.9 - 20.2	55.2 - 79.1	67.0 - 88.1
>30-40	58	26 (44.8%)	40 (69.0%)	24 (41.4%)	21 (36.2%)	15 (25.9%)	18 (31.0%)	12 (20.7%)	45 (77.6%)	48 (82.8%)
		32.0 - 58.4	55.3 - 80.1	28.9 - 55.0	24.3 - 49.9	15.6 - 39.3	19.9 - 44.7	11.6 - 33.7	64.4 - 87.1	70.1 - 91.0
>40-50	19	10 (52.6%)	10 (52.6%)	10 (52.6%)	7 (36.8%)	5 (26.3%)	6 (31.6%)	3 (15.8%)	13 (68.4%)	15 (78.9%)
		29.5 - 74.8	29.5 - 74.8	29.5 - 74.8	17.2 - 61.4	10.1 - 51.4	13.6 - 56.5	4.2 - 40.5	43.5 - 86.4	53.9 - 93.0
>50	11	5 (45.5%)	8 (72.7%)	3 (27.3%)	4 (36.4%)	2 (18.2%)	2 (18.2%)	1 (9.1%)	9 (81.8%)	9 (81.8%)
		18.1 - 75.4	39.3 - 92.7	7.3 - 60.7	12.4 - 68.4	3.2 - 52.2	3.2 - 52.2	0.5 - 42.9	47.8 - 96.8	47.8 - 96.8
p-value^a^		0.3604	0.0190	0.1771	0.0990	0.2352	0.0214	0.0343	0.6047	0.9320

^a^  Chi square test for comparison of groups.

**Table:** (b) Extent of marker elevation in relation to age

		bHCG [x-fold of ULN]			AFP [x-fold of ULN]			LDH [x-fold of ULN]		
Age categories (years)	n (%)	Min	Median (Q1 - Q3)	Max	Min	Median (Q1 - Q3)	Max	Min	Median (Q1 - Q3)	Max
*Nonseminoma*										
<=20	17 (10.1%)	0.41	4.89 (0.94 - 30.23)	11290.83	0.25	5.33 (1.53 - 29.11)	1596.20	0.57	1.07 (0.86 - 1.21)	4.08
>20-30	63 (37.5%)	0.21	2.08 (0.45 - 64.54)	749063.67	0.10	0.89 (0.27 - 12.20)	1290.83	0.55	0.89 (0.75 - 1.04)	17.00
>30-40	58 (34.5%)	0.03	0.65 (0.45 - 12.41)	130974.14	0.13	2.34 (0.60 - 19.54)	1630.46	0.59	0.88 (0.78 - 1.11)	3.81
>40-50	19 (11.3%)	0.34	1.31 (0.41 - 6.78)	113.11	0.13	3.47 (0.62 - 11.83)	5906.58	0.69	1.17 (0.89 - 1.43)	9.32
>50	11 (6.5%)	0.04	0.56 (0.21 - 4.15)	30.00	0.43	2.20 (0.93 - 68.14)	277.50	0.65	0.89 (0.82 - 1.05)	2.71
p-Value^a^			0.0947			0.0498			0.0800	
*All GCT*										
<=20	18 (4.3%)	0.41	4.47 (0.94 - 30.23)	11290.83	0.11	4.93 (1.03 - 29.11)	1596.20	0.57	1.03 (0.84 - 1.21)	4.08
>20-30	86 (20.4%)	0.03	1.52 (0.45 - 25.13)	749063.67	0.08	0.39 (0.22 - 2.86)	1290.83	0.55	0.89 (0.72 - 1.04)	17.00
>30-40	159 (37.7%)	0.03	0.45 (0.41 - 1.76)	130974.14	0.08	0.36 (0.22 - 1.18)	1630.46	0.01	0.89 (0.73 - 1.11)	8.40
>40-50	110 (26.1%)	0.03	0.45 (0.41 - 1.58)	113.11	0.05	0.39 (0.26 - 0.59)	5906.58	0.57	0.89 (0.81 - 1.16)	9.32
>50	49 (11.6%)	0.03	0.45 (0.41 - 1.00)	30.00	0.17	0.47 (0.30 - 0.75)	277.50	0.03	0.89 (0.76 - 1.03)	2.71
p-Value^a^			<.0001			0.0014			0.0960	

^a^  Kruskal Wallis-Test for comparisons of groups; ULN upper limit of norm; min lowest marker level in this group; max highest marker level in this group; Q1 first quartile; Q3 third quartile.

**Table 7:** Frequencies of elevated marker levels in CS1 patients in relation to treatment.

	Seminoma			Nonseminoma			
		bHCG elevated	LDH elevated		bHCG elevated	AFP elevated	LDH elevated
	n	n (%)	n (%)	n	n (%)	n (%)	n (%)
		95% CI [%]	95% CI [%]		95% CI [%]	95% CI [%]	95% CI [%]
Before orchiectomy	201	49 (24.4%)	46 (22.9%)	80	37 (46.3%)	50 (62.5%)	18 (22.5%)
		18.7 - 31.0	17.4 - 29.4		35.2 - 57.7	50.9 - 72.9	14.2 - 33.5
postoperatively	201	15 (7.5%)	33 (16.4%)	80	15 (18.8%)	39 (48.8%)	6 (7.5%)
		4.4 - 12.2	11.7 - 22.4		11.2 - 29.4	37.5 - 60.1	3.1 - 16.2
p-value^a^		<.0001	0.0280		<.0001	0.0009	0.0027
At completion of adjuvant therapy	201	2 (1.0%)	22 (10.9%)	80	1 (1.3%)	1 (1.3%)	10 (12.5%)
		0.2 - 3.9	7.1 - 16.3		0.1 - 7.7	0.1 - 7.7	6.5 - 22.2
p-value^a^		<.0001	0.0004		<.0001	<.0001	0.0736

^a^McNemar Test for comparisons of expression rates with rate before orchiectomy.

**(a):** 

	all GCT patients (>CS1)				nonseminoma patients (>CS1)			
		bHCG elevated	AFP elevated	LDH elevated		bHCG elevated	AFP elevated	LDH elevated
		n (%)	n (%)	n (%)		n (%)	n (%)	n (%)
	(n)	95% CI [%]	95% CI [%]	95% CI [%]	(n)	95% CI [%]	95% CI [%]	95% CI [%]
Time-point 1	141	74 (52.5%)	54 (38.3%)	75 (53.2%)	88	52 (59.1%)	51 (58.0%)	47 (53.4%)
		43.9 - 60.9	30.4 - 46.9	44.6 - 61.6		48.1 - 69.3	47.0 - 68.2	42.5 - 64.0
Time-point 2	141	53 (37.6%)	48 (34.0%)	46 (32.6%)	88	42 (47.7%)	46 (52.3%)	28 (31.8%)
		29.7 - 46.2	26.4 - 42.6	25.1 - 41.1		37.1 - 58.6	41.4 - 62.9	22.5 - 42.7
p-value		<.0001	0.1088	<.0001		0.0124	0.1655	0.0004
Time-point 3	123	26 (21.1%)	30 (24.4%)	45 (36.6%)	84	24 (28.6%)	29 (34.5%)	30 (35.7%)
		14.5 - 29.6	17.3 - 33.1	28.2 - 45.8		19.5 - 39.6	24.7 - 45.8	25.8 - 47.0
p-value		<.0001	<.0001	0.0013		<.0001	<.0001	0.0094
Time-point 4	141	4 (2.8%)	5 (3.5%)	43 (30.5%)	88	4 (4.5%)	4 (4.5%)	30 (34.1%)
		0.9 - 7.6	1.3 - 8.5	23.2 - 38.9		1.5 - 11.9	1.5 - 11.9	24.5 - 45.1
p-value		<.0001	<.0001	0.0113		<.0001	<.0001	0.0113

p-value: McNemar test for comparisons of expression rates; all comparisons relate to time-point 1

time-point1: before orchiectomy

time-point 2: after orchiectomy

time-point 3: after first cycle of chemotherapy (not all patients had chemotherapy)

time-point 4: at completion of therapy.

**Table:** (b) Changes of median marker levels during treatment in all GCT patients with metastases (>CS1)

		bHCG [x-fold of ULN]			AFP [x-fold of ULN]			LDH [x-fold of ULN]		
	N	Min	Median (Q1 - Q3)	Max	Min	Median (Q1 - Q3)	Max	Min	Median (Q1 - Q3)	Max
Time-point 1	141	0.18	1.31 (0.45 - 14.38)	749063.67	0.12	0.59 (0.27 - 3.29)	5906.58	0.03	1.04 (0.88 - 1.42)	17.00
Time-point 2	141	0.03	0.46 (0.41 - 8.59)	357084.27	0.12	0.51 (0.26 - 1.84)	1291.42	0.36	0.89 (0.78 - 1.20)	14.05
Time-point 3	123	0.03	0.45 (0.41 - 0.67)	10728.54	0.10	0.58 (0.36 - 0.97)	2386.53	0.40	0.92 (0.75 - 1.14)	4.68
Time-point 4	141	0.03	0.45 (0.41 - 0.45)	19.13	0.10	0.38 (0.24 - 0.49)	1.78	0.53	0.89 (0.76 - 1.03)	2.17
p-value			<.0001			<.0001			<.0001	

p-value: Friedman Test for comparison of marker levels at four time-points; ULN upper limit of norm; Q1 lower quartile; Q3 upper quartile

time-point 1: before orchiectomy

time-point 2: after orchiectomy

time-point 3: after first cycle of chemotherapy, if applicable; not all patients had chemotherapy

time-point 4: at completion of therapy.

**Table:** (a) Frequencies of elevated marker levels in relapsing patients

		bHCG elevated	AFP elevated	LDH elevated	bHCG and AFP elevated	bHCG and LDH elevated	AFP and LDH elevated	bHCG, AFP and LDH elevated	bHCG or AFP elevated	bHCG, AFP or LDH elevated
	N	n (%)	n (%)	n (%)	n (%)	n (%)	n (%)	n (%)	n (%)	n (%)
		95% CI [%]	95% CI [%]	95% CI [%]	95% CI [%]	95% CI [%]	95% CI [%]	95% CI [%]	95% CI [%]	95% CI [%]
*at diagnosis *										
all patients	422	160 (37.9%)	108 (25.6)	139 (32.9)	67 (15.9%)	73 (17.3%)	46 (10.9%)	30 (7.1%)	201 (47.6%)	251 (59.5%)
		33.3 - 42.8	21.6 - 30.1	28.5 - 37.7	12.6 - 19.8	13.9 - 21.3	8.2 - 14.4	4.9 - 10.1	42.8 - 52.5	54.6 - 64.2
no relapse	390	151 (38.7%)	100 (25.6)	130 (33.3)	61 (15.6%)	70 (17.9%)	43 (11.0%)	28 (7.2%)	190 (48.7%)	235 (60.3%)
		33.9 - 43.8	21.4 - 30.3	28.7 - 38.3	12.3 - 19.7	14.3 - 22.2	8.2 - 14.7	4.9 - 10.3	43.7 - 53.8	55.2 - 65.1
later relapse	32	9 (28.1%)	8 (25.0%)	9 (28.1%)	6 (18.8%)	3 (9.4%)	3 (9.4%)	2 (6.3%)	11 (34.4%)	16 (50.0%)
		14.4 - 47.0	12.1 - 43.8	14.4 - 47.0	7.9 - 37.0	2.5 - 26.2	2.5 - 26.2	1.1 - 22.2	19.2 - 53.2	32.2 - 67.8
p-value^a^		0.2351	0.9363	0.5405	0.6436	0.2157	0.7698	0.8412	0.1184	0.2559
*at relapse*										
yes	27	7 (25.9%)	6 (22.2%)	8 (29.6%)	4 (14.8%)	3 (11.1%)	4 (14.8%)	3 (11.1%)	9 (33.3%)	13 (48.1%)
		11.9 - 46.6	9.4 - 42.7	14.5 - 50.3	4.9 - 34.6	2.9 - 30.3	4.9 - 34.6	2.9 - 30.3	17.2 - 54.0	29.2 - 67.6
p-value^b^		0.5271	0.3173	10.000	0.4142	10.000	0.6547	0.5637	0.4795	0.5271

^a^  Chi square-Test for comparison of marker expression rates among relapsing patients and those without relapse

^b^  Mc-Nemar-Test for comparison of marker expression rates of relapsing patients at different time points.

**Table:** (b) Marker levels of nonseminoma patients with relapse

		bHCG [x-fold of ULN]			AFP [x-fold of ULN]			LDH [x-fold of ULN]		
	n	Min	Median (Q1 - Q3)	Max	Min	Median (Q1 - Q3)	Max	Min	Median (Q1 - Q3)	Max
*at diagnosis*										
not relapsing	157	0.03	1.39 (0.45 - 19.18)	749063.67	0.10	1.93 (0.53 - 12.66)	1630.46	0.55	0.90 (0.77 - 1.11)	17.00
later relapsing	11	0.34	0.67 (0.41 - 16.78)	6347.41	0.24	2.56 (0.36 - 16.79)	5906.58	0.69	0.96 (0.79 - 1.16)	2.39
p-value a			0.8799			0.9030			0.7715	
*at relapse*										
patients with relapse	10	0.34	0.45 (0.41 - 3.28)	229.51	0.14	0.49 (0.39 - 9.54)	49.88	0.62	0.97 (0.88 - 1.06)	1.77

^a^  Mann-Whitney U Test for comparison of median serum marker levels.

**Table 10:** Marker elevation patterns at diagnosis and at relapse.

at diagnosis				at relapse																								
				bHCG	AFP	LDH	bHCG	AFP	LDH	bHCG	AFP	LDH	bHCG	AFP	LDH	bHCG	AFP	LDH	bHCG	AFP	LDH	bHCG	AFP	LDH	bHCG	AFP	LDH	change of marker pattern
(n)	bHCG	AFP	LDH	+	+	+	+	+	-	+	-	+	+	-	-	-	+	+	-	+	-	-	-	+	-	-	-	
2	+	+	+	1			0			0			0			0			0			0			1			1
4	+	+	-	0			1			0			1			1			0			0			1			3
1	+	-	+	0			0			0			0			0			0			0			1			1
2	+	-	-	0			0			0			0			0			0			0			2			2
1	-	+	+	0			0			0			0			0			1			0			0			1
1	-	+	-	1			0			0			0			0			0			0			0			1
4	-	-	+	0			0			0			0			0			0			3			1			1
12	-	-	-	1			0			0			2			0			0			1			8			4

27				3			1			0			3			1			1			4			14			13

**Table 11:** Tumour marker elevation rates in nonseminoma patients – synopsis of studies.

First author	year	n	AFP (%)	bHCG (%)	AFP or bHCG (%)	AFP and bHCG (%)
Szymendera [17]	1983	113	53.1	58.4		

Nørgaard-Petersen [18]	1984	296	52.4	31.1	60	23

Fossa [19]	1987	95			70.5	

Bassoulet [20]	1988	95	66	55	80	43

Fargeot [21]	1989	111	64.0	53.6		

Kausitz [22]	1992	205	72.7	30.2	79.0	23.9

Javadpour [23]	1992	226	65.0	56.2	84.1	

Kulkarni [24]	1993	166			68.7	

Germa-Lluch [25]	2002	852	70.0	52.9		44.0

Neumann [26]	2011	73	66.7			

Rothermundt [27]	2018	107	55.1	55.1		

Dieckmann [28]	2019	187	59.7	63.6	78	

**Table 12:** Tumour marker elevation rates in seminoma – synopsis of studies.

First author	year	n	bHCG (%)	LDH (%)
Kuber [29]	1982	98	20	

Szymendera [17]	1983	61	16.4	

Nørgaard-Petersen [18]	1984	307	6.8	

Kratzik [30]	1988	120	23	

Dieckmann [31]	1989	83	12	

Fossa [32]	1989	105	32	46

Javadpour [23]	1992	160	9	

Rüther [33]	1994	106	30.2	

Weissbach [34]	1997	726	35	34

Germa-Lluch [25]	2002	434	21	-

Neumann [26]	2011	72	18.8	

Sanchis Bonet [35]	2011	58	29.3	

Rothermundt [27]	2018	192	18.8	20.3

Dieckmann [28]	2019	302	31.8	29.9

## Data Availability

The raw data of the present work can be freely accessed at https://www.albertinen.de/krankenhaeuser/albertinen_krankenhaus/zentren_kliniken_institute/Urologie_uroonkologie/forschung_studien.
